# Effect of Normalization on Statistical and Biological Interpretation of Gene Expression Profiles

**DOI:** 10.3389/fgene.2012.00160

**Published:** 2013-05-31

**Authors:** Shaopu Qin, Jinhee Kim, Dalia Arafat, Greg Gibson

**Affiliations:** ^1^School of Biology, Georgia Institute of TechnologyAtlanta, GA, USA

**Keywords:** microarray analysis, normalization, variance component analysis, eSNP

## Abstract

An under-appreciated aspect of the genetic analysis of gene expression is the impact of post-probe level normalization on biological inference. Here we contrast nine different methods for normalization of an Illumina bead-array gene expression profiling dataset consisting of peripheral blood samples from 189 individual participants in the Center for Health Discovery and Well Being study in Atlanta, quantifying differences in the inference of global variance components and covariance of gene expression, as well as the detection of variants that affect transcript abundance (eSNPs). The normalization strategies, all relative to raw log2 measures, include simple mean centering, two modes of transcript-level linear adjustment for technical factors, and for differential immune cell counts, variance normalization by interquartile range and by quantile, fitting the first 16 Principal Components, and supervised normalization using the SNM procedure with adjustment for cell counts. Robustness of genetic associations as a consequence of Pearson and Spearman rank correlation is also reported for each method, and it is shown that the normalization strategy has a far greater impact than correlation method. We describe similarities among methods, discuss the impact on biological interpretation, and make recommendations regarding appropriate strategies.

## Introduction

Normalization is one of the most vexing issues associated with the analysis of functional genomic datasets such as gene expression, metabolomic, and methylation profiles. Much consideration has been given to methods for extracting appropriate probe summary measures from raw microarray, Affymetrix, or Illumina fluorescence intensities, which is the first step in normalization. Even so, given an appropriately pre-processed dataset (Schmid et al., 2010), statistical methods for hypothesis testing that utilize both frequentist and Bayesian approaches are robust and well-established (Efron et al., 2001; Quackenbush, 2002; Bolstad et al., 2003; Allison et al., 2006). It is however less well appreciated just how large the impact of these initial post-probe level data processing steps can be, and these are the subject of this study. This is particularly important where the desire exists to make adjustments for covariates that are thought *a priori* to globally impact a large proportion of the measurements (Qiu et al., 2005; Leek and Storey, 2007), a prime example being leukocyte cell counts in studies of peripheral blood gene expression.

The most commonly utilized normalization methods treat all of the measurements jointly, and are generally variations on approaches to centering the data distributions or equilibrating the variances. Centering approaches most simply include mean or median centering to adjust for overall differences in concentration (perhaps due to slight variation in the amount of sample, or efficiency of the labeling), but ANOVA approaches can also be used if it is suspected that certain groups of samples are likely to have different distributions (Dabney and Storey, 2007; Mason et al., 2010). In all cases, hypothesis testing evaluates differential abundance, usually on a log scale. Variance normalization by contrast effectively evaluates differences in rank order (Durbin et al., 2002), since efforts to ensure that all of the samples have similar variance will tend to equilibrate absolute differences in abundance. The simplest approaches are to convert the measures to *z*-scores by dividing through by the sample standard deviation following centering (Colantuoni et al., 2002), or to perform the hypothesis testing directly on the ranks (Breitling and Herzyk, 2005), both of which obviate any ability to infer fold changes due to the relevant effects. Interquartile range (IQR) normalization forces the distributions to have the same values for the 25th and 75th percentiles (Geller et al., 2003), while the most aggressive approach, quantile normalization (QNM; Bolstad et al., 2003; Hansen et al., 2012), equilibrates all ranks: by assigning each measure the mean value across samples for each rank, all samples are converted to the identical distribution albeit with different ordering of measures. QNM has become the standard method in many circumstances, and is certainly appropriate where the assumption is that only a small number of measures differ among samples and hence that variation in the distributions is mostly technical noise that should be removed. However, in many biological circumstances a large fraction of measures vary systematically due to regulatory mechanisms that create extensive covariance, and we demonstrate herein how QNM can alter the biological signal (see Leek et al., 2010).

Recently, attention has turned to methods that treat different measures unequally, recognizing that both technical and biological factors are both likely to impact only a subset of all of the measures in the samples. Intensity-dependent effects, for example, are often removed by lowess transformation (Yang et al., 2002) that adjusts for biologically unrealistic trends for probes at different intensities to be affected differentially by covariates such as the dye used for labeling cDNA. More generally, it should be recognized that technical factors such as RNA quality may not affect all transcripts equally, and that biological factors such as sex or cell counts in complex tissues, will impact thousands of measures but by no means all. Such global influences can be identified by principal component or similar analysis (Quackenbush, 2001; James et al., 2004; Leek and Storey, 2007), or if known can be modeled directly in the normalization step prior to hypothesis testing, and either removed entirely or adjusted for by forcing samples with similar values of the variable to adopt more similar measurements only for those measurements that are affected (Listgarten et al., 2010; Mecham et al., 2010). This is an intuitively appealing approach that is just beginning to gain traction following the development of open source algorithms, including Supervised Normalization of Microarrays (SNM; see also Stegle et al., 2010) that facilitate the complex calculations involved.

The objective of this study was to quantitatively evaluate the impact of nine different normalization approaches on a new dataset that we are analyzing for the purpose of measuring the impact of clinical covariates on peripheral blood gene expression in healthy adults. Full description of the study will be published elsewhere, as we only describe the influence of four biological variables (gender, ethnicity, age, and BMI) as well as blood cell counts, and two technical variables that commonly impact gene expression studies, namely date of hybridization and RNA quality. The nine methods are: raw log2 transformation (RAW); mean centering (MEA); IQR variance normalization; gene-by-gene adjustment for date and RNA integrity with variance standardization(dr3); date and RNA integrity adjustment followed by mean centering of each sample profile (DRM); further adjustment for the absolute number of lymphocytes, monocytes, neutrophils (LMN), erythrocytes, and platelets; fitting the first 16 principal components to the data (PCA); QNM; and implementation of the SNM procedure to remove technical and blood cell count effects and adjust for a series of biological covariates simultaneously. We document the widespread impact of these methods, draw conclusions regarding similar aspects of their performance, and discuss the implications for interpretation of hypothesis testing.

## Materials and Methods

This study reanalyzes peripheral blood transcriptome data from the Center for Health Discovery and Well Being (CHDWB) study of 189 health adults from Atlanta, GA, USA. Gene expression profiles were generated with Illumina HT-12 V3 bead arrays, and the raw data is available at GEO as study accession number GSE35846. We consider here the abundance of transcripts measured with 14,343 probes that are consistently detected across multiple datasets of peripheral blood samples, which for this study were obtained from Tempus tubes (Applied Biosystems, Foster City, CA, USA) that preserve whole blood RNA. Whole genome genotypes were measured using Illumina OmniQuad arrays. After quality filtering, we retained 34,548 common variants (MAF > 0.05) on Chromosome 6 for the eQTL analyses. All statistical analyses were performed in JMP Genomics v5 (SAS Institute, Cary, NC, USA).

Normalization was performed as follows. Each of the data files is available from the authors’ website at http://www.gibsongroup.biology.gatech.edu/supplementary-data. *RAW* refers to the average bead fluorescence intensity for each probe obtained directly from Bead Studio without background subtraction, with log base 2 transformation but no adjustment across arrays. *MEA* refers to mean centering of the RAW profiles for each sample, namely an additive shift on the log base 2 scale that ensures that the mean value is the same for each individual, but the shape and variance of each profile is not adjusted. Technical batch and RNA quality effects were adjusted giving rise to the *dr3* profiles, by fitting an ANOVA to each probe with fixed effects of hybridization date and Bioanalyzer RNA Integrity Number (RIN) and then standardizing the residuals to yield *z*-score gene expression measures (that is, each gene has a mean of zero and variance of 1 across the 189 individuals). Date has five levels where between 24 and 48 samples (three to six chips each with eight arrays) were hybridized on each of 5 days in a 1 month period in July/August 2010. RIN was categorized with three levels, namely poor quality (RIN < 7), moderate quality (7 < RIN < 8), or good quality (RIN > 8). *DRM* refers to profiles obtained by mean centering of the dr3 profiles, which ensures that there is no bias in the overall distribution of transcripts with relatively low or high expression in each individual, as expected biologically. The dr3 profiles were subject to an alternate transformation adjusting for blood cell counts, giving rise to the *LMN* profiles by fitting probe-specific multiple linear regression with counts of Lymphocytes, Monocytes, Neutrophils, Erythrocytes, and Platelets (all measured directly using a standard CBC panel on each sample), and retaining the residuals.

Two types of variance transformation were performed. *IQR* refers to the InterQuartile Range, namely the distribution of each RAW log base 2 profile adjusted to ensure that the range between the 25th and 75th percentile values is 1 and that these are the same for each sample. This produces more similar variance structure than the MEA transform, while also ensuring that all arrays have similar means. *QNM* refers to quantile normalization, which is a density-adjusted rank ordering. For each sample, each probe is ranked according to intensity and then the average intensity of each rank is computed. The probe is assigned that average value, resulting in identical overall distributions.

The other two normalizations considered here are SNM and PCA. *SNM* refers to supervised normalization of microarrays and was performed using the *R* package of that name from Bioconductor (Mecham et al., 2010). For the model reported here, we fit and removed effects of Date, RIN, and the absolute counts of seven cell types (lymphocytes, monocytes, neutrophils, erythrocytes, platelets as well as eosinophils, and basophils), and also adjusted for various blood parameters (albumin, alkaline phosphatase, alanine aminotransferase, vitamin B12, bilirubin, blood urea nitrogen, calcium, chloride, cholesterol, HDL, and LDL, creatinine, blood CO_2_, vitamin D, ferritin, globulin, glucose, iron capacity, mean corpuscular hemoglobin, potassium, triglycerides, thyroxine, and the inflammatory cytokines IL6, IL8, and TNFα). *PCA* refers to profile residuals after fitting a multiple linear regression with each of the first 16 principal components of expression of all 14,434 probes across the 189 samples. These 16 PC explain 61.5% of the variance cumulatively; all remaining PC explain less than 1% each.

Variance component analysis was performed using the Basic Expression Workflow routine in JMP Genomics. The first five principal components of the total gene expression dataset were computed, and then an average of the proportion of the trait explained by each of these five PC, weighted by their contributions to the total gene expression, was computed. Immuno-informative axes of variation are defined as the first PC of 10 definitive genes for each of seven axes described in (Preininger et al., submitted). Volcano plots are simply *x*–*y* plots of significance as the negative logarithm of the *p*-value against fold-change in gene expression between the indicated groups.

## Results

### Data distribution

**Figure 1:**
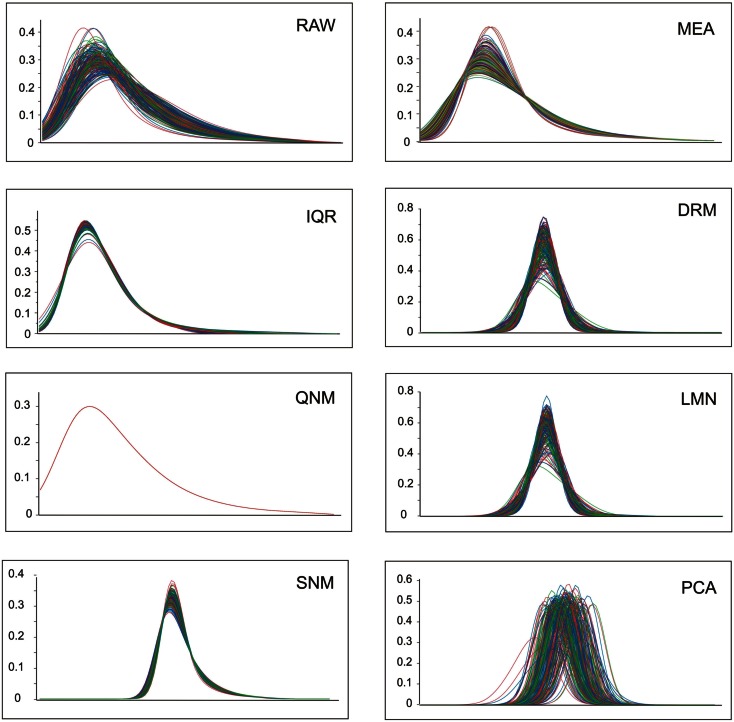
**Profile distributions after nine modes of normalization**. Each plot shows the frequency distribution of transcripts at increasing levels of expression along the *x*-axis (the units are removed, since these are not comparable between methods). Colors represent normal weight (blue), heavy (green), or obese (red) individuals.

A visual representation of the impact of the normalization on data distributions is provided in Figure 1. Log transformation of the RAW data results in approximately normal distributions of all samples, albeit with a left-shifted peak due to most transcripts having low to moderate abundance with a long tail of higher abundance transcripts. The mean and variance of these distributions may or may not be correlated with biological and technical covariates. Since the three colors here represent normal weight, overweight, and obesity, there is evidently no clear overall impact of these BMI classes on the gene expression profiles. MEA ensures that overall abundance effects are removed, while IQR further squeezes the distributions into more similar profiles. The next three methods all result from gene-by-gene fitting of covariates using ANOVA and/or linear regression as appropriate, followed by standardization of the measures. The dr3 plot fitting date and RNA integrity results in *z*-score distributions with different means (similar to those in the PCA plot in Figure 1), so these are further adjusted by a mean centering to produce the DRM panel. Similarly, LMN produces mean-centered *z*-score distributions after fitting the number (similar results are obtained after fitting the proportion, not shown) of the five major blood cell types that *a priori* are likely to impact global gene expression profiles. The bottom row shows the effect of the more aggressive normalization procedures. PCA objectively removes most of the sources of covariance without regard to the source; the resultant standardized distributions are further mean-centered for all subsequent analyses. The QNM plot shows how QNM forces all samples to the same overall distribution, and clearly the SNM procedure is almost as effective at adjusting both the mean and variance of the distributions, but in a more experimentally (less statistically) motivated manner.

### Covariance

**Figure 2:**
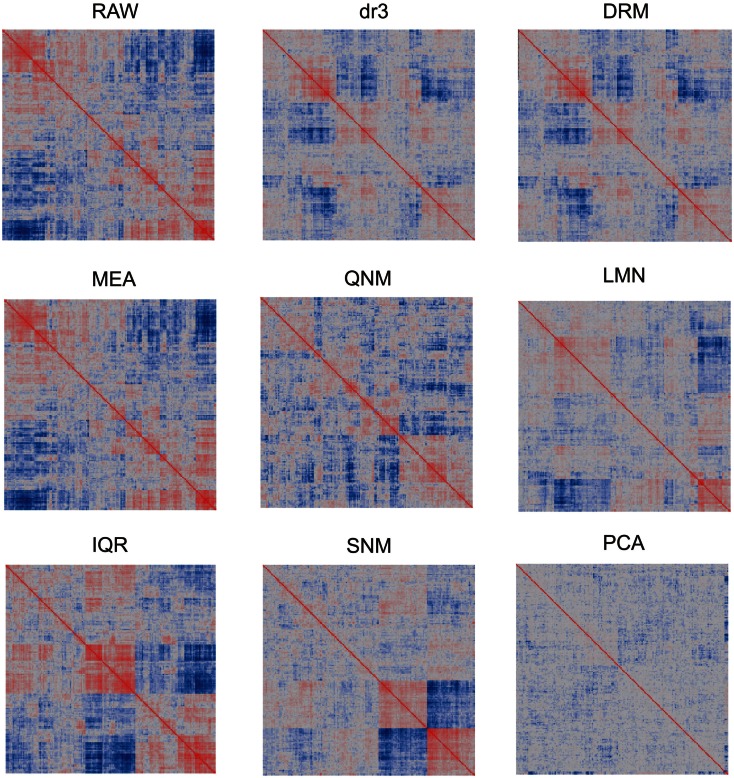
**Heatmaps showing pair-wise similarity of arrays**. Each plot shows the correlation coefficient for the correlation coefficients of each gene expression in each array with that in the paired array. Values range from −1 (dark blue) to +1 (dark red). Blocks of color indicate that arrays in those sectors are less or more similar to one another. Each plot is symmetrical about the diagonal.

In light of the dramatic impact of normalization on the data distributions, it is to be expected that patterns of covariance of gene expression might also be affected. We visualize this in two ways. Figure 2 shows the overall similarity of distributions as a heat map of the pair-wise correlation coefficients of each array with each other array. Note that the precise ordering of arrays is not the same for each normalization. As expected, the RAW and MEA correlation clusters are identical, since the correlation coefficients are not affected by additive adjustment of the grand mean, and these are similar to the two straight variance transforms (IQR and QNM) since there has been no change in the ranking of transcript abundance. dr3/DRM and LMN clearly impact the covariance structure as they adjust for artificial correlations induced by the technical factors and shared gene expression within cell types. PCA almost completely removes any covariance, certainly removing some shared biological regulation in the process. The SNM leaves a novel pattern of covariance that in theory improves on LMN by also adjusting for other sources of biological variation such as gender and ethnicity. The two clusters of individuals at the bottom right represent the extremes for PC1 after SNM normalization, but no single trait that was included in the normalization model explains this separation of expression profile types.

**Figure 3:**
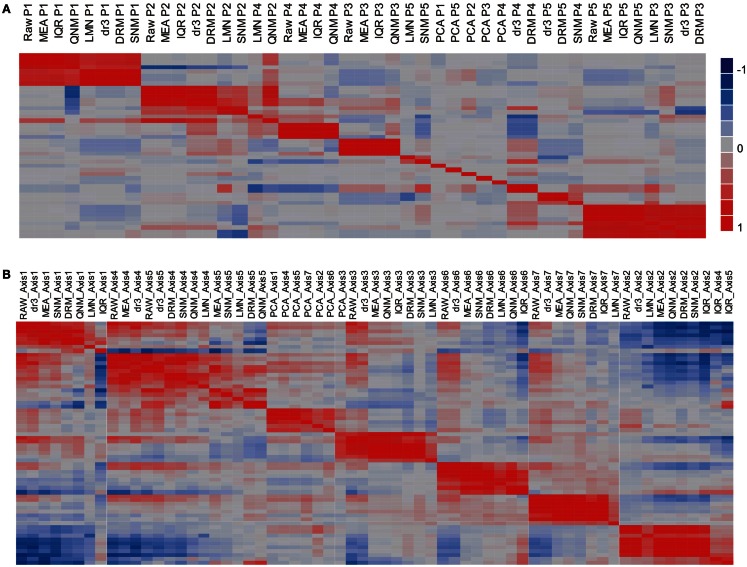
**Similarity of principal components (A) and immuno-informative axis scores (B)**. The heat maps show the correlation coefficient across all 189 samples for each PC axis, where the order of the rows is the same as the order of the columns. **(A)** Comparison of the first five PC shows that PC1 is generally highly correlated across normalization strategies, as is PC2, but that the lower PC fall into different clusters. **(B)** By contrast, the primary axis of covariance of genes representing seven common axes of immunologically informative variation (Risso et al., 2011) are generally well conserved across all eight normalization strategies (excepting PCA).

The potential impact of the normalization procedures on biological inference is perhaps more readily appreciated by evaluating the impact on the major principal components of residual gene expression variation, as shown in Figure 3A, a heat map of the pair-wise correlations between the first five Principal Components across the nine normalization strategies. Strikingly, PC1 remains highly correlated (*r* > 0.5) across all of the normalization methods with the exception of PCA (which was expected to remove this factor). There are two groups with almost identical PC1 eigenvalues: RAW, MEA, IQR, QNM, and dr3/DRM, LMNEP, SNM. This is as expected, and shows that cell counts have very little impact on the major axis of variation in peripheral blood. Surprisingly, the QNM procedure does not separate its PC2 from PC1 assessed with the other procedures.

The next three PC are correlated to varying degrees with neutrophil, monocyte, and lymphocyte counts in particular. The two methods that adjust for cell numbers, LMN, and SNM, alter PC2 substantially, with the result that strong correlations are observed between their PC3 scores and PC2 with the other methods. Since the minor PC only explain 2–4% of the variance each, slight changes in variance component can change their relative rank, as well as their sign (which has no biological meaning). It is also striking that although PC are by definition orthogonal within a normalization, across normalizations they generally pick up overlapping components of covariance so that a single PC in one analysis can significantly correlate with multiple PC in another analysis: thus, PC4 under DRM evidently captures covariance that contributes to each of PC2, PC4, and PC5 under MEA normalization. It is also important to note that the impact of QNM is biologically difficult to interpret as the PCs show the least similarity with those derived from the other methods. By contrast, SNM removes the cell abundance effects as expected and generally shows a covariance structure that is a composite of fitting cell types and technical factors.

### Variance components and gene-specific linear regression

Mirroring the changes in correlation structure, normalization can have a dramatic impact on the covariance of the principal components of variation with traits of interest. Here we consider just four: Gender, Ethnicity, and partitioning of BMI into low, normal/overweight, and obese reflecting their proportions in the population, and of Age into four levels, namely young, 40s, 50s, and older. A weighted sum of the proportion of variance of each that is captured by the first five principal components is shown in Table 1. Fitting the technical covariates Date and RNA Integrity (RIN3) in the DRM, LMN, and SNM models removes these contributions efficiently. Most of the methods suggest that similar amounts of gene expression variation are explained by three of the traits (BMI has little effect overall), although SNM apportions almost twice as much variance to Gender and Ethnicity as do the other methods, while fitting the blood cell counts removes the Gender component since the blood counts differ slightly between men and women. RAW, MED and IQR are essentially identical since they have little impact on the covariance structure, but QNM enhances the Gender relative to Ethnicity contribution. PCA has essentially removed all of the biological contributions that result in covariance.

**Table 1:** **Variance component analyses**.

Normalization	Date	RIN	Age	BMI	Gender	Ethnicity	Residual
RAW	43.7	5.1	1.5	0.1	3.3	2.8	43.5
MEA	43.7	5.1	1.5	0.1	3.3	2.8	43.5
dr3	0	0	2.0	0.5	2.7	4.8	90.0
DRM	0	0	2.0	0.5	2.7	4.8	90.0
IQR	43.3	4.9	1.6	0.1	3.1	3.0	44.2
LMN	0	0	1.7	0.3	0.1	3.3	94.5
QNM	38.5	7.9	1.7	0.2	5.0	3.5	45.2
SNM	0	0.2	1.8	0.9	5.9	7.4	83.8
PCA	2.5	0.7	0.5	0.1	1.2	4.3	90.7

*The table reports the weighted average of the percentage of variation explained by the first five principal components of gene expression, for the indicated variables. Samples were hybridized on five different days in the July and August 2010, and RIN refers to three categorical levels of RNA integrity number (<7, 7–8, >8). Age was modeled as a categorical variable with four levels (<40, 40–50, 50–60, >60); BMI as a categorical variable with three levels (<25, 25–30, >30); and Ethnicity has three levels (Caucasian, African American, Asian)*.

Similarly, gene-by-gene modeling of the association between transcript abundance and continuous trait measures is a strong function of normalization. Table 2 shows the number of transcripts at the per-trait Bonferroni multiple comparison adjusted threshold (*p* < 0.00007) after fitting a multivariate ANOVA with AGE, BMI, Gender, and Ethnicity. There is a threefold range in the total number of highly significant associations detected, with the least observed for analysis of the RAW data, and the most overall for QNM. Age associations that are partially correlated with the technical covariates in this sample are not detected after QNM, while SNM facilitates enhancement of the BMI effect, possibly at the expense of Gender and Ethnicity effects. The simple expedient of mean centering is at least as effective as the mild IQR variance adjustment. Also indicated is the impact of failing to adjust for array effects after fitting the technical and cell number covariates (compare the dr3 and DRM rows), since overall profile differences are mildly correlated with the biological factors. Once again, fitting the first 16 PCA completely removes most trait associations. Similar trends are observed at less stringent significance thresholds.

**Table 2:** **Trait associations**.

Normalization	Age	BMI	Gender	Ethnicity	Total
RAW	4	0	40	59	103
MEA	4	0	75	159	238
dr3	16	0	34	89	139
DRM	7	0	64	198	269
IQR	3	0	58	151	212
LMN	2	2	15	101	120
QNM	3	0	90	201	294
SNM	13	5	38	140	196
PCA	0	0	3	2	5

*The table reports the total number of associations detected between Probe-level expression, and the indicated traits. Age was modeled as a categorical variable with four levels (<40, 40–50, 50–60, >60); BMI as a categorical variable with three levels (<25, 25–30, >30); and Ethnicity has three levels (Caucasian, African American, Asian)*.

**Figure 4:**
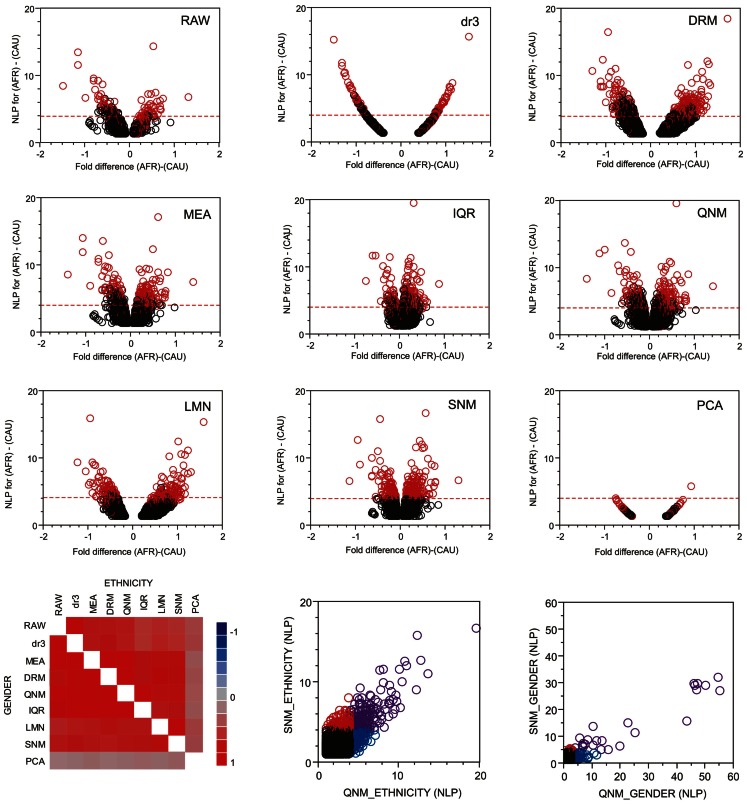
**Volcano plots of significance and comparison of thresholds**. Volcano plots contrast significance as the negative logarithm of the *p*-value against differential expression, in this case for all genes with NLP > 1.3 (nominal *p* < 0.05) in the contrast of African American and Caucasian samples in the CHDWB study. Red circles are genes that are significant at NLP > 4 in the SNM normalization, and the horizontal dashed red line shows this threshold for each method. In general, highly significant contrasts are significant in all methods, but this is not necessarily the case for the Gender comparison where Quantile normalization (QNM) over-represents the gender effect relative to all other methods. The heat map at the bottom left shows the pair-wise correlation of estimated effect sizes for all 14,343 probes for each normalization comparison, for ethnicity above the diagonal, and gender below it.

The good news in this analysis is that there is extensive overlap in the most significant transcripts identified after each normalization approach. Figure 4 shows volcano plots of significance against difference in abundance for the African American versus Caucasian contrast following each normalization approach, with the significant genes from SNM normalization indicated in red. There is wide variation in the shapes of the plots, with IQR and LMN showing poor and strong separation of up- and down-regulated genes respectively. It should be noted that the fold-difference axis is presented on the log2 scale in the former, and on *z*-scores for the latter. All points in the dr3 plot align on a simple curve, since all genes have the same variance after standardization (re-centering in DRM adds variance back, resulting in the more typical volcano plot). The correlation in the *p*-values for both Ethnicity and Gender is high across all eight methods (excluding PCA, bottom left panel), but note that there is a strong over-estimation of the significance of the Gender effect in QNM relative to SNM (bottom right panel, blue circles) and that many genes are only called significant for Ethnicity with either procedure (red and blue circles). Given the wide variety of significance thresholds adopted for taking genes forward for downstream processing steps such as Gene Ontology analysis, it is not clear whether normalization has as larger effect than simply setting the threshold for inclusion of genes in downstream analysis. The impact will largely be study-specific, but these analyses indicate that it will rarely be negligible.

### eSNP analysis

In order to evaluate the effect of normalization on the ability to detect regulatory influences of locally acting SNPs on transcript abundance, we next performed so-called cis-eSNP analysis (Cheung et al., 2005; Stranger et al., 2005; GuhaThakurta et al., 2006), using chromosome 6 for illustrative purposes. This chromosome contains the MHC which is enriched for hundreds of cis-eQTL association, but otherwise is no different from the other autosomes. The total number of SNPs associated with expression of a target transcript located within 250 kb on either side of the probe, and the number of independent eSNP-probe associations are shown in Table 3 (for illustrative purposes simply defined as one eSNP-probe association per gene). Data is presented both for Pearson correlation with the normalized abundance measures, and Spearman rank correlations. At the accepted 10^−8^ threshold for genome-wide significance, there are anywhere from 39 to 88 independent associations, depending on the normalization approach. Relaxation of significance thresholds recovers most of the associations detected in the more sensitive analyses. There are few instances where a highly significant eSNP effect observed with one analysis is not seen at least nominally with the others, indicating that the normalization does not generally induce spurious associations. Yet the more than twofold difference in detection rate is striking, and would have potential consequences where the purpose is to compare regulatory profiles across conditions and/or tissues.

**Table 3:** **eSNP analyses**.

Normalization	Pearson correlation				Spearman rank correlation	
	Total (NLP 8)	Cis (NLP 5)	Cis (NLP 8)	Probes (NLP 8)	Cis (NLP 8)	Probes (NLP 8)
RAW	552	1183	411	39	324	36
MEA	1082	2009	743	77	703	71
dr3	627	1362	455	44	407	46
DRM	959	2150	761	87	747	77
IQR	935	1708	603	71	565	73
LMN	484	1281	439	44	394	44
QNM	1211	2288	842	88	791	81
SNM	969	2084	825	86	821	81
PCA	602	1563	585	73	505	74

*The Table reports the total number of associations detected between 34,548 Chromosome 6 SNPs and 732 Chromosome 6 Probes, respectively including total (trans and cis) associations at NLP 8; just cis-associations at NLP 5 or NLP 8 (defining cis as eSNPs within 250 kb of the probe); the number of independent probes with eSNPs at NLP 8 (all using Pearson correlation with the transcript abundance); and then the cis-associations and number of independent probes at NLP 8 using Spearman rank correlation*.

An important result is that PCA efficiently removes almost all trans-associations (585 out of 602 associations at 10^−8^ are in cis, compared with just two thirds (603 of 935) for IQR eSNP associations), but recovers most cis-associations detected by the “better” normalization strategies. It does not however improve on those strategies, since both SNM and QNM uncover more cis-associations both individually and at the probe level. It has been argued that PCA should remove all sources of environmental variance, hence enhancing the genetic contribution to expression variation and leading to the detection of more cis-eSNPs. This however clearly does not happen, but we also note that the number of PCA that are fit can also influence the results, as discussed in (Stegle et al., 2010). One possibility is that analyses of whole blood are biased toward detecting effects that are fairly consistent across cell types, and that aggressive removal of all covariance disrupts the matrix of cellular contributions. Under controlled culture conditions where individual cell lines are profiled, PCA may well be the most efficient normalization procedure, but our analysis cautions that this may not always be the case. SNM results in slightly fewer associations than QNM normalization, but this loss of power is offset by the improved ability to detect trait correlations described above and by a reduction in the number of trans-associations that are more likely to be false positives. Finally, Pearson and Spearman rank correlations are universally similar with slightly fewer associations detected with the rank-based association method, but normalization strategy having the far greater impact on eSNP detection.

## Discussion

Both the technology and statistical theory for gene expression profiling have advanced considerably in the past decade, but there remains considerable inconsistency and confusion about the most appropriate way to analyze complex datasets. Much attention is paid to significance testing, with methods ranging from adjusted *t*-tests (Tusher et al., 2001) and ANOVA (Ayroles and Gibson, 2006), through shrinkage estimation to account for differences in variance (Yang and Churchill, 2007) and the optimal discovery procedure (Storey et al., 2007) utilizing the power of shared regulation, to empirical Bayesian approaches (e.g., Cao et al., 2009; Guindani et al., 2009). However, all of these procedures are only as good as the input data set, which in turn needs to be processed in a suitable manner to remove spurious bias. The purpose of this contribution is to illustrate how processing, downstream of initial data acquisition (Schmid et al., 2010), can impact statistical and biological inference with a typical dataset of almost 200 microarrays incorporating several biological variables and the usual technical batch (date) and RNA quality effects. It is sometimes assumed that RNA-Seq will obviate such technical issues (Wang et al., 2009), but it creates similar and novel concerns of its own (Robinson and Oshlack, 2010; Risso et al., 2011; Hansen et al., 2012), and in any case all of the issues we raise regarding normalization of biological biases will be just as important for sequence-based as array-based studies.

### Absolute and relative differential expression

The first major decision that must be taken in data normalization is whether to optimize the strategy to detect differences in absolute transcript levels, or relative abundance. If large numbers of transcripts are affected by some treatment, then it is possible that they show only minor changes in rank order: in this case, the overall shape of the profile is an important feature of the data, and analyses are preferred that retain the individual profile variance. For example, an environmental effect may be to spread the entire distribution over a larger range. In other circumstances, especially where only a relatively small number of probes are expected to change (for example, in a contrast of mutants, or with genomic DNA hybridizations), it may be the relative abundance that is of most interest (Yang et al., 2002). Any procedure that adjusts the variance of the profile shifts the analysis toward rank order comparisons. In the most aggressive case of QNM, all variation in range or shape of the overall profile is deliberately removed, and along with it the ability to detect absolute changes irrespective of rank order. On the other hand, there is something intuitively appealing about equilibrating the profile shapes, and where there is no obvious biological source of the similarity of subsets of profiles it can be argued that technical variance is the more likely cause, and thence that it should be removed.

Whichever decision is made, the analyses reported here highlight how strong the effect on inference may be. Simple mean (or median) centering has no effect on the covariance structure, but it is an effective first step toward removing pervasive biases across the transcriptome and demonstrably increases the variance explained by the first five principal components, increases the number of transcript associations with biological covariates, and increases the power of eSNP analysis. Variance transforms such as IQR and QNM can have effects ranging from subtle to substantive: in this particular dataset, QNM produces stronger trait associations, but there is some suggestion that it perturbs the covariance structure in ways that may over-estimate some effects and under-estimate others. Fitting Principal Components may be desirable in situations where technical variance is suspected to obscure relatively weak biological signals (for example, among cultured cell lines), but in many situations the major PC actually capture important biological sources of variance (for example, strong cancer profiles, tissue effects, drug responses, plant growth regimes) and so their removal is not advisable.

Perhaps more importantly, it is very clear that neither centering nor variance adjustments do anything about confounding effects of either technical or biological covariates: the batch effects of hybridization on different dates continue to account for over a third of the total transcript variance. In an earlier study of peripheral blood profiles (Mason et al., 2010), we showed how dramatically gene-specific technical variance adjustment impacted our ability to identify a major biological source of covariance between mothers and their newborn children. Such technical effects are pervasive in datasets in GEO (Barrett et al., 2011) and are almost impossible to avoid, unless the budget allows for fully randomized replication, which is rarely the case. The two choices are to accept that they add noise, and ignore them, or attempt to remove them by more aggressive strategies that include them as covariates in the normalization model.

### Technical and biological adjustment variables

The simplest strategy for modeling covariates is to perform gene-specific linear modeling, taking the residuals forward for downstream analysis. This has the effect of removing the terms that are included in the regression or ANOVA. Here, we illustrated two steps of adjustment. First the technical factors (Date, and RNA integrity) were modeled as fixed categories, with three levels of RIN. There is a danger in over-fitting by binning samples into small classes that may also make the analysis susceptible to outliers, but this can generally be controlled by plotting the principal components as a function of the categorical levels – and in turn, such data visualization can be used to help define the appropriate bins. Second, we also performed linear adjustment for the abundance of five types of contributing blood cell by multivariate regression of each transcript on the five measures. Inspection of the beta coefficients can be used to assess which transcripts are strongly affected by cell abundance, and it is clear that at least a quarter of the transcriptome varies as a function of cell types. Recognizing this, though, underscores the motivation for transcript-specific adjustment: centering and variance transforms treat all transcripts equally, but they are not all equally affected by the technical covariates.

When using these procedures, we chose to standardize the residuals so that the abundance measures are in *z*-scores, namely standard deviations of one around a mean of zero for each transcript independently. It is important also to post-process the scores to account for array effects, namely to re-center the residuals, otherwise some arrays systematically have low or high values, which is probably biologically implausible – it is unlikely that any one individual will have expression below the average for all transcripts, for example. The difference between dr3 and DRM shows that this process restores the association of transcripts with traits and the ability to detect eSNPs, and it does so without the concern that associations may be driven more by covariance between biological and technical factors. This mode of adjustment can be extended to include latent, or surrogate, variables (Leek and Storey, 2007), including principal components that are suspected to represent unwanted sources of error. Clearly fitting a large number of principal components runs the risk of absorbing all of the biological factors, so is not advised as a generic strategy, though there will be circumstances where it is justified and appropriate.

A more computationally demanding, but intuitively appealing, approach is to simultaneously fit the covariates to all probes simultaneously. This is the approach taken by the SNM of microarrays and related procedures (Listgarten et al., 2010; Mecham et al., 2010; Stegle et al., 2010). The notion is that all transcripts that are jointly affected by some factor will tend to co-vary in a detectable and adjustable manner. Either, the factor is known to be a source of error (like date or RNA quality) and is removed essentially as above, or it is recognized as something to be adjusted for, in which case the profiles of those genes that are co-regulated are forced toward more similar distributions without removing the effect. Depending on the objectives of the analysis, the same factor may be removed or adjusted for. In our SNM example, we chose to remove the cell count effects, and since they are correlated to some extent with BMI, age, and gender, this has the effect of altering the association of individual transcripts with the traits, for example increasing the proportion of variance associated with Age and BMI, but decreasing that with ethnicity. Supervised analyses are then by their nature not only study-specific, but also analysis-specific, and as such are perhaps the most powerful approach for specific hypothesis testing. Implicitly, they emphasize that there is no single correct way to normalize a dataset.

### Assessing performance

Nevertheless, it is desirable to have metrics of performance that might guide the choice of the most appropriate normalization strategy. Probably the most useful is repeatability, which can be frustratingly elusive in microarray datasets. In fact, this issue was the motivation for the development of the SNM approach: faced with four parallel trauma datasets that yielded incomparable *p*-value distributions following standard normalization procedures, Storey and co-workers (Mecham et al., 2010) recognized that covariance with biological and technical factors was responsible and reasoned that fitting them jointly may solve the problem. Indeed, careful SNM resulted in all four independent datasets yielding similar levels of enrichment for small *p*-values, and focused the analysis on a consistent set of biomarkers (Desai et al., 2011). For sufficiently large datasets that can be divided into two or more subsets, comparison of the significance levels across random subsets using different normalization strategies should help discover the most suitable strategy, and this may include adjustment for different subsets of biological covariates.

Another metric could be the consistent detection of expected patterns of covariance. Figure 3A shows that standard principal components tend not to replicate consistently across normalization strategies. However, more biologically sound patterns of covariance may do so. For example, we recently described how seven highly conserved axes of variation explain the majority of the covariance of peripheral blood transcription (Preininger et al., submitted). Just 10 blood-informative transcripts can be used to generate PC scores that are highly correlated with hundreds to thousands of transcripts associated with each axis. Figure 3B shows that for the most part, these axis scores are independent of the normalization method, with the most divergent scores observed for the LMN strategy of fitting cell counts, consistent with differential abundance of some cell types contributing to the axis scores. IQR also interferes slightly with the assessment of just the first axis, but notably the QNM and SNM procedures are in good agreement with one another and with the simpler normalization strategies. This implies that for the investigation of the structure of certain biological pathways and networks, normalization may be less critical than it is for significance assessment and variance partitioning.

In summary, our consideration of nine different normalization strategies highlights how the first phase of analysis of gene expression datasets can influence various aspects of statistical and in turn biological inference. Similar arguments can be made with respect to other types of high throughput data including RNA-Seq, methylation and chromatin profiling, and comparative genome hybridization. Where the dataset is known to be affected by multiple biological factors, or suspected to be perturbed by hidden variables, it will generally pay to explore and compare diverse strategies, searching both for consistency, as well as differences that may themselves point to interesting biology.

## Conflict of Interest Statement

The authors declare that the research was conducted in the absence of any commercial or financial relationships that could be construed as a potential conflict of interest.

## References

[B1] Allison D. B. Cui X. Page G. P. Sabripour M. (2006). Microarray data analysis: from disarray to consolidation and consensus. Nat. Rev. Genet. 7, 55–65 10.1038/nrg1869 16369572

[B2] Ayroles J. F. Gibson G. (2006). Analysis of variance of microarray data. Meth. Enzymol. 411, 214–233 10.1016/S0076-6879(06)11011-3 16939792

[B3] Barrett T. Troup D. B. Wilhite S. E. Ledoux P. Evangelista C. Kim I. F. Tomashevsky M. Marshall K. A. Phillippy K. H. Sherman P. M. Muertter R. N. Holko M. Ayanbule O. Yefanov A. Soboleva A. (2011). NCBI GEO: archive for functional genomics data sets-10 years on. Nucleic Acids Res. 39, D1005–D1010 10.1093/nar/gkq848 21097893 PMC3013736

[B4] Bolstad B. M. Irizarry R. A. Astrand M. Speed T. P. (2003). A comparison of normalization methods for high density oligonucleotide array data based on variance and bias. Bioinformatics 19, 185–193 10.1093/bioinformatics/19.2.185 12538238

[B5] Breitling R. Herzyk P. (2005). Rank-based methods as a non-parametric alternative of the T-statistic for the analysis of biological microarray data. J. Bioinform. Comput. Biol. 3, 1171–1189 10.1142/S0219720005001442 16278953

[B6] Cao J. Xie X. J. Zhang S. Whitehurst A. White M. A. (2009). Bayesian optimal discovery procedure for simultaneous significance testing. BMC Bioinformatics 10:5 10.1186/1471-2105-10-5 19126217 PMC2628883

[B7] Cheung V. G. Spielman R. S. Ewens K. G. Weber T. M. Morley M. Burdick J. T. (2005). Mapping determinants of human gene expression by regional and genome-wide association. Nature 437, 1365–1369 10.1038/nature04244 16251966 PMC3005311

[B8] Colantuoni C. Henry G. Zeger S. Pevsner J. (2002). SNOMAD (Standardization and NOrmalization of MicroArray Data): web-accessible gene expression data analysis. Bioinformatics 18, 1540–1541 10.1093/bioinformatics/18.11.1540 12424128

[B9] Dabney A. R. Storey J. D. (2007). Normalization of two-channel microarrays accounting for experimental design and intensity-dependent relationships. Genome Biol. 8, R44 10.1186/gb-2007-8-3-r44 17391524 PMC1868928

[B10] Desai K. H. Tan C. S. Leek J. T. Maier R. V. Tompkins R. G. Storey J. D. (2011). Dissecting inflammatory complications in critically injured patients by within-patient gene expression changes: a longitudinal clinical genomics study. PLoS Med. 8:e1001093 10.1371/journal.pmed.1001093 21931541 PMC3172280

[B11] Durbin B. P. Hardin J. S. Hawkins D. M. Rocke D. M. (2002). A variance-stabilizing transformation for gene-expression microarray data. Bioinformatics 18(Suppl. 1), S105–S110 10.1093/bioinformatics/18.suppl_1.S105 12169537

[B12] Efron B. Tibshirani R. Storey J. D. Tusher V. (2001). Empirical Bayes analysis of a microarray experiment. J. Am. Stat. Assoc. 96, 1151–1160 10.1198/016214501753382129

[B13] Geller S. C. Gregg J. P. Hagerman P. Rocke D. M. (2003). Transformation and normalization of oligonucleotide microarray data. Bioinformatics 19, 1817–1823 10.1093/bioinformatics/btg245 14512353

[B14] GuhaThakurta D. Xie T. Anand M. Edwards S. W. Li G. Wang S. S. Schadt E. E. (2006). Cis-regulatory variations: a study of SNPs around genes showing cis-linkage in segregating mouse populations. BMC Genomics 7:235 10.1186/1471-2164-7-235 16978413 PMC1618400

[B15] Guindani M. Müller P. Zhang S. (2009). A bayesian discovery procedure. J. R. Stat. Soc. Series B Stat. Methodol. 71, 905–925 10.1111/j.1467-9868.2009.00714.x 20694043 PMC2914327

[B16] Hansen K. D. Irizarry R. A. Wu Z. (2012). Removing technical variability in RNA-seq data using conditional quantile normalization. Biostatistics 13, 204–216 10.1093/biostatistics/kxr054 22285995 PMC3297825

[B17] James J. Chen J. J. Delongchamp R. Tsai C.-A. Hsueh H. Sistare F. Thompson K. Desai V. G. Fuscoe J. C. (2004). Analysis of variance components in gene expression data. Bioinformatics 20, 1436–1446 10.1093/bioinformatics/bth118 14962916

[B18] Leek J. T. Scharpf R. B. Corrada-Bravo C. Simcha D. Langmead B. Johnson W. E. Geman D. Baggerly K. Irizarry R. A. (2010). Tackling the widespread and critical impact of batch effects in high-throughput data. Nat. Rev. Genet. 11, 733–739 10.1038/nrn2943 20838408 PMC3880143

[B19] Leek J. T. Storey J. D. (2007). Capturing heterogeneity in gene expression studies by surrogate variable analysis. PLoS Genet. 3:e161 10.1371/journal.pgen.0030161 PMC1994707 17907809

[B20] Listgarten J. Kadie C. Schadt E. E. Heckerman D. (2010). Correction for hidden confounders in the genetic analysis of gene expression. Proc. Natl. Acad. Sci. U.S.A. 107, 16465–16470 10.1073/pnas.1002425107 20810919 PMC2944732

[B21] Mason E. Tronc G. Nones K. Matigian N. Kim J. Aronow B. J. Wolfinger R. D. Wells C. Gibson G. (2010). Maternal influences on the transmission of leukocyte gene expression profiles in population samples from Brisbane, Australia. PLoS ONE 5:e14479 10.1371/journal.pone.0014479 21217831 PMC3013110

[B22] Mecham B. H. Nelson P. S. Storey J. D. (2010). Supervised normalization of microarrays. Bioinformatics 26, 1308–1315 10.1093/bioinformatics/btq118 20363728 PMC2865860

[B23] Qiu X. Brooks A. I. Klebanov L. Yakovlev N. (2005). The effects of normalization on the correlation structure of microarray data. BMC Bioinformatics 6:120 10.1186/1471-2105-6-120 15904488 PMC1156869

[B24] Quackenbush J. (2001). Computational analysis of microarray data. Nat. Rev. Genet. 2, 418–427 10.1038/35076576 11389458

[B25] Quackenbush J. (2002). Microarray data normalization and transformation. Nat. Genet. 32, 496–501 10.1038/ng1032 12454644

[B26] Risso D. Schwartz K. Sherlock G. Dudoit S. (2011). GC-content normalization for RNA-Seq data. BMC Bioinformatics 12:480 10.1186/1471-2105-12-480 22177264 PMC3315510

[B27] Robinson M. D. Oshlack A. (2010). A scaling normalization method for differential expression analysis of RNA-seq data. Genome Biol. 11, R25 10.1186/gb-2010-11-s1-p25 20196867 PMC2864565

[B28] Schmid R. Baum P. Ittrich C. Fundel-Clemens K. Huber W. Brors B. Eils R. Weith A. Mennerich D. Quast K. (2010). Comparison of normalization methods for Illumina BeadChip HumanHT-12 v3. BMC Genomics 11:349 10.1186/1471-2164-11-349 20525181 PMC3091625

[B29] Stegle O. Parts L. Durbin R. Winn J. (2010). A Bayesian framework to account for complex non-genetic factors in gene expression levels greatly increases power in eQTL studies. PLoS Comput. Biol. 6:e1000770 10.1371/journal.pcbi.1000770 20463871 PMC2865505

[B30] Storey J. D. Dai J. Y. Leek J. T. (2007). The optimal discovery procedure for large-scale significance testing, with applications to comparative microarray experiments. Biostatistics 8, 414–432 10.1093/biostatistics/kxl019 16928955

[B31] Stranger B. E. Forrest M. S. Clark A. G. Minichiello M. J. Deutsch S. Lyle R. Hunt S. Kahl B. Antonarakis S. E. Tavaré S. Deloukas P. Dermitzakis E. T. (2005). Genome-wide associations of gene expression variation in humans. PLoS Genet. 1:e78 10.1371/journal.pgen.0010078 16362079 PMC1315281

[B32] Tusher V. G. Tibshirani R. Chu G. (2001). Significance analysis of microarrays applied to the ionizing radiation response. Proc. Natl. Acad. Sci. U.S.A. 98, 5116–5121 10.1073/pnas.091062498 11309499 PMC33173

[B33] Wang Z. Gerstein M. Snyder M. (2009). RNA-Seq: a revolutionary tool for transcriptomics. Nat. Rev. Genet. 10, 57–63 10.1038/nrm2594 19015660 PMC2949280

[B34] Yang H. Churchill G. (2007). Estimating p-values in small microarray experiments. Bioinformatics 23, 38–43 10.1093/bioinformatics/btm143 17077100

[B35] Yang Y. H. Dudoit S. Luu P. Lin D. M. Peng V. Ngai J. Speed T. P. (2002). Normalization for cDNA microarray data: a robust composite method addressing single and multiple slide systematic variation. Nucleic Acids Res. 30, e15 10.1093/nar/30.10.2251 11842121 PMC100354

